# Monosodium glutamate delivered in a protein-rich soup improves subsequent energy compensation

**DOI:** 10.1017/jns.2014.15

**Published:** 2014-08-13

**Authors:** Una Masic, Martin R. Yeomans

**Affiliations:** School of Psychology, University of Sussex, Brighton BN1 9QH, UK

**Keywords:** Monosodium glutamate, Intake, Appetite, Hunger, Protein, ED, energy density, MSG, monosodium glutamate, MSG+, MSG added, MSG–, no added MSG

## Abstract

Previous research suggests that monosodium glutamate (MSG) may have a biphasic effect on appetite, increasing appetite within a meal with its flavour-enhancing effect, but enhancing subsequent satiety due to its proposed role as a predictor of protein content. The present study explored this by assessing the impact of a 450 g soup preload differing in MSG concentration (1 % MSG added (MSG+) or no MSG (MSG–)) and nutrient content (low-energy control or high-energy carbohydrate or high-energy protein) on rated appetite and *ad libitum* intake of a test meal in thirty-five low-restraint male volunteers using a within-participant design. Protein-rich preloads significantly reduced intake at the test meal and resulted in more accurate energy compensation than did carbohydrate-rich preloads. This energy compensation was stronger in the MSG+ protein conditions when compared with MSG+ carbohydrate conditions. No clear differences in rated appetite were seen in MSG or the macronutrient conditions alone during preload ingestion or 45 min after intake. Overall, these findings indicate that MSG may act to further improve energy compensation when provided in a protein-rich context.

Monosodium glutamate (MSG) is a flavour enhancer that improves the savoury experience of foods and is the prototypical chemical associated with the ‘umami’ taste^(^^1^^–^^3^^)^. Because of its role as a flavour enhancer, it was initially believed that MSG increased appetite and intake^(^^4^^–^^11^^)^. However, initial enhanced intake tends to decrease over time^(^^8^^,^^12–^^14^^)^ and recent research suggests that MSG may also delay hunger recovery in the short term too, particularly when in combination with a food rich in protein^(^^15^^)^.

So why might any appetite-enhancing effects of MSG be short-lived? One explanation is that appetite is stimulated by MSG during the course of the meal (reducing satiation), but then suppressed during the post-ingestive stage to delay later intake (enhancing satiety). This suggestion is based on the idea that our ability to detect MSG evolved as a means of detecting the presence of protein in foods^(^^1^^)^ and regulating protein consumption^(^^16^^,^^17^^)^. There is clear evidence that protein enhances satiety more effectively than does either carbohydrate^(^^18^^–^^20^^)^ or fat^(^^21^^,^^22^^)^ when delivered acutely as a preload^(^^23^^,^^24^^)^ and longer term in the diet^(^^25^^–^^27^^)^. Recent data suggest that the satiating effects of protein are in part due to the sensory characteristics of protein-rich foods acting to enhance post-ingestive satiety^(^^28^^)^, and umami could be one of the critical cues generating these sensory–nutrient interactions. Indeed, the umami taste has been linked to the satiating effects of protein in infants^(^^29^^,^^30^^)^ and may explain why intake of MSG meals over time remains stable despite increases in palatability. A previous study assessing the time course of changes in rated appetite over 120 min after consumption of a fixed volume of soup (high-energy carbohydrate-rich or protein-rich or low-energy control) with and without added MSG also supports these findings. Hunger decreased less after MSG soup intake (consistent with the stimulation of appetite through flavour enhancement), but hunger recovery was slower in the MSG protein-rich condition compared with the no-MSG protein-rich condition which was not seen for MSG in a low-energy or carbohydrate-rich context^(^^15^^)^. However, another study has found no effect of MSG and protein on satiety when measured at a test meal^(^^31^^)^. This discrepancy might be due to the previous study^(^^15^^)^ relying on measures of rated appetite at predefined time points whilst other research has assessed intake at *ad libitum* test meal sessions^(^^31^^)^.

Given this ambiguity in the literature about the role of MSG in appetite control, the present research examined the effects of a soup preload differing in specific macronutrient ratios (high-energy soups further enhanced with additional protein and carbohydrate contrasted with a low-energy control) either with MSG added (MSG+) or no MSG (MSG–) on appetite and intake at a subsequent *ad libitum* meal. A preload meal interval of 45 min was used as this was found to be the optimum time for differences in hunger and fullness ratings between MSG+ and MSG– conditions in our recent study^(^^15^^)^. Although MSG is not a nutritive substance, its potential influence on satiation and satiety may modify the response to nutrients, particularly when in combination with high ratios of a satiating nutrient such as protein. It was hypothesised that the flavour-enhancing effects of MSG would mean less of a decrease in hunger when consuming a fixed portion of the MSG+ versions regardless of nutrient content, but that hunger would recover more slowly, and consequently that test-meal intake would be less in the protein-rich than carbohydrate-rich preload conditions relative to the control, with MSG+ enhancing the satiating effects of protein.

## Methods

### Design

The study used a within-participant design to examine the effects of consumption of a fixed soup preload differing in specific nutrient ratios/energy (high-energy carbohydrate-rich or protein-rich or low-energy control) and MSG content (1 % (w/w) MSG (MSG+) or no MSG (MSG–)). Preload condition order was balanced using a Williams square design^(^^32^^)^.

### Participants

A total of thirty-six low-restraint males initially participated in the research but one participant failed to complete all sessions. The thirty-five remaining participants (mean age: 21 (sd 0·4) years, range 18–28 years; mean BMI: 22 (sd 0·5) kg/m^2^, range 18–33 kg/m^2^) were students at the University of Sussex. Sample size was determined from our earlier study^(^^15^^)^ with effect size based on the maximal difference in rated hunger between the protein preload with and without added MSG, indicating that thirty-six participants would be required. Exclusion criteria included smoking, being on medications, a history of diabetes, diagnosed eating disorders, allergies or dietary intolerances to the foods used. Also, those with high restraint scores (ratings above 7 on the Three-Factor Eating Questionnaire^(^^33^^)^) were excluded, as restrained individuals may not be representative of general eating behaviour^(^^34^^,^^35^^)^. Prospective participants were emailed with details of the study disguised as ‘assessing the effects of food on motor skills’ to minimise demand effects of the experimental manipulation. Written informed consent was given before participation and participants were paid £60 on completion. The study was conducted in accordance with the standards expressed in the Helsinki Declaration and was approved by the University of Sussex ethics committee.

### Test food

#### Control breakfast

Breakfast on all test days consisted of 80 g cereal (Crunchy Nut Cornflakes; Kellogg's), 200 g semi-skimmed milk (Sainsbury's plc) and 200 g orange juice (Sainsbury's plc) (total 2107 kJ (503·6 kcal)). These quantities were established based on UK Food Standard Agency guidelines for male breakfast consumption^(^^36^^)^.

#### Soup preloads

All flavour and energy manipulations used the same low-energy density (ED) control soup, which was a carrot and spice soup containing carrots (Frozen Baby Carrots; Sainsbury's plc), onions, celery, olive oil (Medium Flavour Olive Oil; Sainsbury's plc), spice mixture (Garam Masala, Schwartz) and water (see Masic &Yeomans^(^^15^^)^). Portion size was fixed at 450 g, as this has been established as an adequate portion for males^(^^5^^)^ and was successful in our previous study^(^^15^^)^. Energy content was enhanced by the addition of 52 g/450 g portion maltodextrin (dextrose equivalent: 15·3; Cargill) in the carbohydrate soup and 17·86 g/450 g maltodextrin (Cargill) combined with 36 g/450 g whey protein isolate (MyProtein UK) to the protein soup. 1 % (w/w) MSG (Ajinomoto) was added to all MSG+ soup conditions. Base soup formulation followed extensive pilot testing to formulate a novel soup low in MSG, and which was rated as moderately pleasant to allow for enhancement by MSG. Pilot testing was also carried out on the energy and macronutrient soup combinations used (see Masic &Yeomans^(^^15^^)^).

The high-ED conditions contained approximately 750 kJ (180 kcal) more per portion than the low-ED condition (carbohydrate: 743·7 kJ (177·5 kcal), protein: 771·1 kJ (184·3 kcal)). The small energy difference between the high-ED carbohydrate and protein conditions was due to efforts to minimise the impact of maltodextrin on sweetness in the carbohydrate condition. All nutritional information can be found in Table 1.

**Table 1.** Nutritional composition of soup preloads (per 100 g)

	Low-energy control	High-energy carbohydrate	High-energy protein
Carbohydrate (g)	3·1	13·6	6·5
Protein (g)	0·4	0·3	7·7
Fat (g)	1·6	1·3	1·5
Carbohydrate (% energy)	44	81	38
Protein (% energy)	6	2	45
Fat (% energy)	50	17	19
Energy			
kJ	117·2	282·0	288·3
Kcal	28·0	67·4	68·9

#### Ad libitum *meal*

The two-course *ad libitum* lunch comprised of pasta in tomato sauce followed by ice cream. Due to an unexpected change in formulation of the pasta sauce by the manufacturer part way through the study, two versions of the meal had to be used but each participant was only tested with one version. Both versions used 250 g cooked pasta (Conchiglie Pasta; Sainsbury's plc). This was combined with 250 g of pasta sauce (Tomato and Basil Sauce; Sainsbury's plc) for version 1, giving a 500 g served portion (total 2273·6 kJ (543·4 kcal)), but since the reformulated sauce had a higher energy density, 200 g of sauce was used for version 2 to ensure a similar energy density across test meals providing a 450 g portion (total 2093·7 kJ (500·4 kcal)). In all, fifteen participants were tested with version 1 and twenty with version 2. Analyses of sensory ratings and intake of the test meal depending on the served version found a significant difference in rated saltiness between the two versions (*F*(5,165) = 2·78; *P* = 0·02) but no other ratings differed significantly, including pleasantness (*F*(5,165) = 0·73; NS). Intake did not differ significantly between those consuming version 1 and version 2 (*F*(5,165) = 0·85; NS) and when test meal version was included as a covariate, no differences in pasta meal intake was apparent (MSG × test meal version: *F*(1,33) = 2·57, NS; MSG × condition × test meal version: *F*(2,66) = 1·34, NS). The served dessert was 150 g of vanilla ice cream (Carte D'Or; Unilever; total 1318 kJ (315 kcal)).

### Computerised data collection

Sensory, hedonic and appetite ratings were tracked using the Sussex Ingestion Pattern Monitor (SIPM version 2·0·13; University of Sussex), which is comprised of a digital balance linked to a computer. Participants were asked to complete appetite, sensory, hedonic and mood ratings using digital visual analogue scales by the SIPM. All ratings were presented as sentences (‘How <word> do you feel?’) with a left-hand anchor reading ‘Not at all <word > ’ (coded as 0) and a right-hand anchor reading ‘As <word> as I have ever felt/experienced’ (coded as 100). Instructions on how to use the scale were presented to participants before each evaluation to ensure compliance. Participants registered their selection by pressing ‘Rating Complete’. Presentation order for each group of visual analogue scale ratings was randomised.

When participants were presented with the fixed preload or relevant course they completed a taste test of the food (familiar, pleasant, salty, savoury, strong and sweet for the preload, the same excluding sweetness for the pasta and excluding salty and savoury for the dessert). Following this, appetite ratings of hunger, fullness and thirst after tasting the relevant course and at the end of the course were completed. At the *ad libitum* lunch participants were given a portion of the food with instructions to ‘Please eat as much (pasta/ice cream) as you like until you feel comfortably full’. Additional (refill) portions of the course being consumed were prompted by the SIPM with an on-screen message and alert sound which instructed participants to call the experimenter.

### Procedure

The research took place over six non-consecutive sessions at the Ingestive Behaviour Unit (IBU) at the University of Sussex. Participants were asked to consume nothing but water from 23·00 hours the night before each testing session and were provided with the control breakfast at pre-arranged times (from 09·00 hours to 10·30 hours) across testing days. Participants were free to leave the IBU after breakfast with instructions to consume nothing but water and returned after 3 h for the soup preload.

**Fig. 1.**
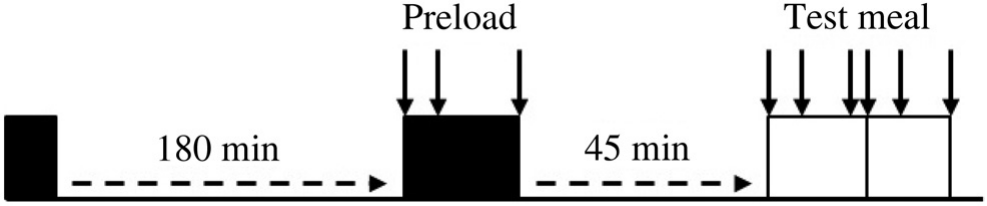
Graphical representation of the timings of the preload and *ad libitum* meals and the appetite ratings made on each test day. ↓, Appetite rating made; ■, fixed meal; □, *ad libitum* meal.

For all consumption trials (preload and test meal) participants were first asked to complete a bogus motor skills task (the star motor task, which consisted of tracing the outline of a star with their non-dominant hand) in compliance with the cover story. Both the preload (which ran from 12·00 hours to 13·30 hours) and main course sessions started with mood and appetite ratings followed by serving of the relevant course. Taste test and appetite ratings were completed after tasting each course (preload, main course and dessert) with further appetite ratings when each course was completed. The test meal was provided 45 min after the soup as this was the time when differences in hunger were maximal between the protein-rich preloads with and without added MSG^(^^15^^)^. For the pasta course a portion of the pasta in sauce was served with refills provided in 500 g (or 450 g) portions after approximately 450 g (or 400 g) consumption whilst a portion of ice cream was served with additional 100 g portions provided after 100 g ice cream consumption. This ensured that food was always present on the plate to prevent normative external cues such as an empty plate from influencing meal intake^(^^37^^,^^38^^)^. The session ended after the final set of mood and appetite ratings. A graphical representation is present in Fig. 1. At the end of sessions 1–5, participants were free to leave but their height and weight was recorded and they were debriefed before payment on the final test day.

### Data analysis

Test meal intake (energy consumed) was contrasted using two-way 3 × 2 repeated-measures ANOVA with soup type (control, protein or carbohydrate) and MSG condition (MSG+ or MSG–) as variables. Intake was also analysed across the whole test meal and by individual courses (savoury course and dessert) to determine whether preload conditions made an impact on intake overall or differed by course. Additional analyses were conducted to determine energy compensation (COMPX) at the test meal after high-energy (protein-rich or carbohydrate-rich) preloads across MSG conditions (MSG+ or MSG–). COMPX values were calculated by subtracting test meal intake in the relevant control (low-energy) MSG+ or MSG– condition from the corresponding protein or carbohydrate (high-energy) MSG+ or MSG– condition and expressing this value as a percentage of the actual difference in soup preload energy between low- and high-energy preloads (protein: 770 kJ (184 kcal); carbohydrate: 741 kJ (177 kcal)). The resulting COMPX scores were contrasted using two-way 2 × 2 repeated-measures ANOVA. To control for repeat testing, test order was included as a factor in all analyses.

Satiation analyses (pre-meal, post-taste and post-course) were conducted for each eating episode over time using 3 × 2 × 3 repeated-measures ANOVA assessing appetite (hunger and fullness) for the preload and separate 3 × 2 × 5 repeated-measures ANOVA assessing appetite for the test meal due to the hypothesised effects of MSG increasing hunger during the preload but maintaining satiety over the test meal duration. A separate 3 × 2 repeated-measures ANOVA analysing the preload effects over 45 min post-ingestion was also carried out to assess the hypothesis of a slower return of hunger in protein-rich MSG+ conditions, as found previously^(^^15^^)^. Hunger and fullness variables during preload satiation were transformed to track the more subtle changes from baseline over the eating episode. Sensory evaluations of the soup and test meal were analysed using repeated-measures two-way 3 × 2 ANOVA. In cases of violated sphericity, Greenhouse Geisser values (ε ≤ 0·75) were adopted. In cases of violated Greenhouse Geisser assumptions (ε ≥ 0·75), Huynh–Feldt values were reported. Effect sizes are reported for specific effects using Pearson's correlation coefficient. Data are shown for all thirty-five participants.

## Results

### Test meal intake

As expected, energy intake at the test lunch varied between the three soup preloads (*F*(1·75,59·55) = 4·58; *P* = 0·02) with significantly less energy consumed after the protein-rich soup relative to control (*F*(1,34) = 7·47, *P* = 0·01, *r* 0·42; Table 2). However, although there was a tendency for lower energy consumption in MSG+ relative to MSG– in both the protein-rich and carbohydrate-rich, but not control, conditions (Table 2), these effects were not significant, nor did any effects of added MSG emerge from analysis of the two courses of the meal separately. An order × condition interaction effect was found for intake of the savoury (*F*(10,58) = 2·41; *P* = 0·02) and sweet (*F*(10,58) = 2·05; *P* = 0·04) course, with inspection of the data across test sessions suggesting that intake was highest in the first session after which consumption was adjusted to the nutrients ingested in the preload.

**Table 2.** Energy intake of an *ad libitum* meal after a soup preload (low-energy control, high-energy carbohydrate or high-energy protein) with (MSG+) or without (MSG–) added monosodium glutamate (Mean values with their standard errors)

	Low-energy control				High-energy carbohydrate				High-energy protein			
	MSG–		MSG +		MSG–		MSG +		MSG–		MSG +	
	Mean	sem	Mean	sem	Mean	sem	Mean	sem	Mean	sem	Mean	sem
Pasta												
kJ	1994·1	149·0	2022·6	157·7	1926·3	128·9	1905·4	127·2	1735·5	113·8	1625·9	110·0
Kcal	476·6	35·6	483·4	37·7	460·4	30·8	455·4	30·4	414·8	27·2	388·6	26·3
Ice cream												
kJ	908·3	79·5	928·0	81·6	920·9	77·0	867·3	80·3	897·5	68·6	846·0	67·8
Kcal	217·1	19	221·8	19·5	220·1	18·4	207·3	19·2	214·5	16·4	202·2	16·2
Total intake												
kJ	2902·4	194·6	2950·6	192·5	2847·3	161·1	2772·7	171·1	2633·0	157·7	2471·9	146·0
Kcal	693·7	46·5	705·2	46	680·5	38·5	662·7	40·9	629·3	37·7	590·8	34·9

### Compensation for preload energy

**Fig. 2.**
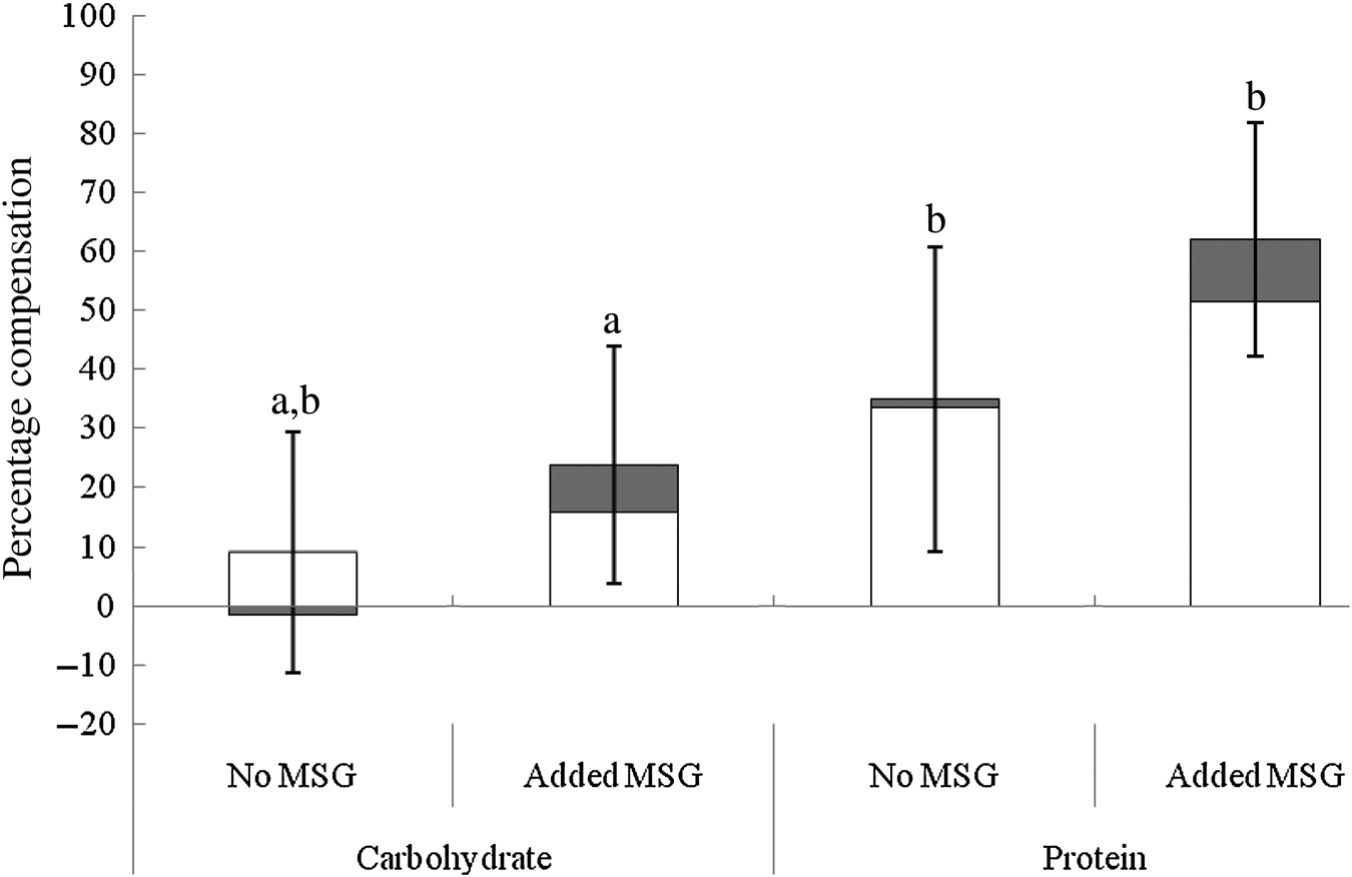
Energy compensation at an *ad libitum* test meal (pasta main course (□) and ice cream dessert (■)) after fixed consumption of high-energy carbohydrate and high-energy protein soup preloads with and without added monosodium glutamate (MSG). Values are means, with standard errors represented by vertical bars. ^a,b^ Mean values with unlike letters were significantly different (*P* ≤ 0·05; within-subjects Bonferroni-corrected contrasts).

Overall energy compensation at the test meal was significantly better in the protein than carbohydrate condition (*F*(1,34) = 4·19, *P* = 0·05, *r* 0·33; Fig. 2). Notably, the small effects of added MSG on lunch intake translated into significantly better compensation for added energy in the protein-rich soup, with 62 % compensation in the protein MSG+ condition compared with only 24 % compensation in the carbohydrate MSG+ condition (*F*(1,34) = 5·45; *P* = 0·03; *r* 0·37). This effect was largely driven by differences in savoury course intake (*F*(1,34) = 5·63; *P* = 0·02; *r* 0·38) with 51 % compensation in pasta intake in the MSG+ protein condition but only 16 % in the carbohydrate MSG+ condition. No significant differences in compensation were found when comparing carbohydrate and protein MSG– conditions overall (*F*(1,34) = 1·21; NS; *r* 0·04).

### Rated hunger and fullness during the preload and test meal

#### Preload satiation and satiety

There were no significant spurious differences in rated hunger (*F*(2,68) = 0·06; NS) or fullness (*F*(2,68) = 0·68; NS) before the soup was tasted. Thus, change from baseline hunger and fullness were analysed to assess the influence of MSG manipulations when the soup was first tasted (assessing the appetiser effect^(^^4^^)^) and immediately after consuming the soup preload (assessing effects on satiation). A significant condition × MSG interaction (*F*(2,68) = 4·10; *P* = 0·02) in hunger ratings immediately after tasting the soup indicated that hunger increased after tasting the MSG+ control and carbohydrate soups but surprisingly decreased after tasting in the MSG+ protein condition whilst in the MSG– conditions, hunger decreased after control and carbohydrate conditions but increased after tasting the protein-rich soup (*F*(1,34) = 5·70, *P* = 0·02, *r* 0·38; Table 3). No significant effects of soup preload on hunger (*F*(2,68) = 0·31; NS) or fullness (*F*(2,68) ≤ 0·001; NS) were found immediately after soup intake across conditions, indicating that differences in nutrient content did not affect satiation. Satiety analyses post-preload to pre-lunch revealed an increase in hunger (*F*(1,34) = 38·30; *P* ≤ 0·001) and decrease in fullness (*F*(1,34) = 59·55; *P* ≤ 0·001) over the 45 min period as expected. However, there was no effect of MSG and no MSG × condition interaction on hunger (MSG: *F*(1,34) = 0·87, NS; condition × MSG interaction: *F*(1·7,57·2) = 0·21, NS) or fullness (MSG: *F*(1,34) ≤0·001, NS; condition × MSG interaction: *F*(1·8,57·3) = 0·33, NS) in contrast to what was found previously^(^^15^^)^.

**Table 3.** Change from baseline visual analogue scale appetite ratings for three versions of soups (low-energy control, high-energy carbohydrate, and high-energy protein) with (MSG+) and without (MSG–) added monosodium glutamate (Mean values with their standard errors)

		Low-energy control				High-energy carbohydrate				High-energy protein			
		MSG–		MSG +		MSG–		MSG +		MSG–		MSG +	
Rating	Time	Mean	sem	Mean	sem	Mean	sem	Mean	sem	Mean	sem	Mean	sem
Hunger	Taste	–4·2	3·3	1·1	1·3	–3·0	2·2	0·1	1·5	2·0	1·1	–3·4	2·1
	Post-preload	–29·2	4·0	–27·2	3·4	–27·4	3·6	–25·0	3·7	–24·8	4·4	–28·3	4·0
Fullness	Taste	1·9	2·0	1·5	1·4	2·8	2·3	2·8	1·8	2·1	1·5	3·5	2·4
	Post-preload	32·7	4·6	32·3	4·1	32·9	4·0	32·1	3·6	32·2	3·7	32·8	3·9

#### Test meal satiation

**Fig. 3.**
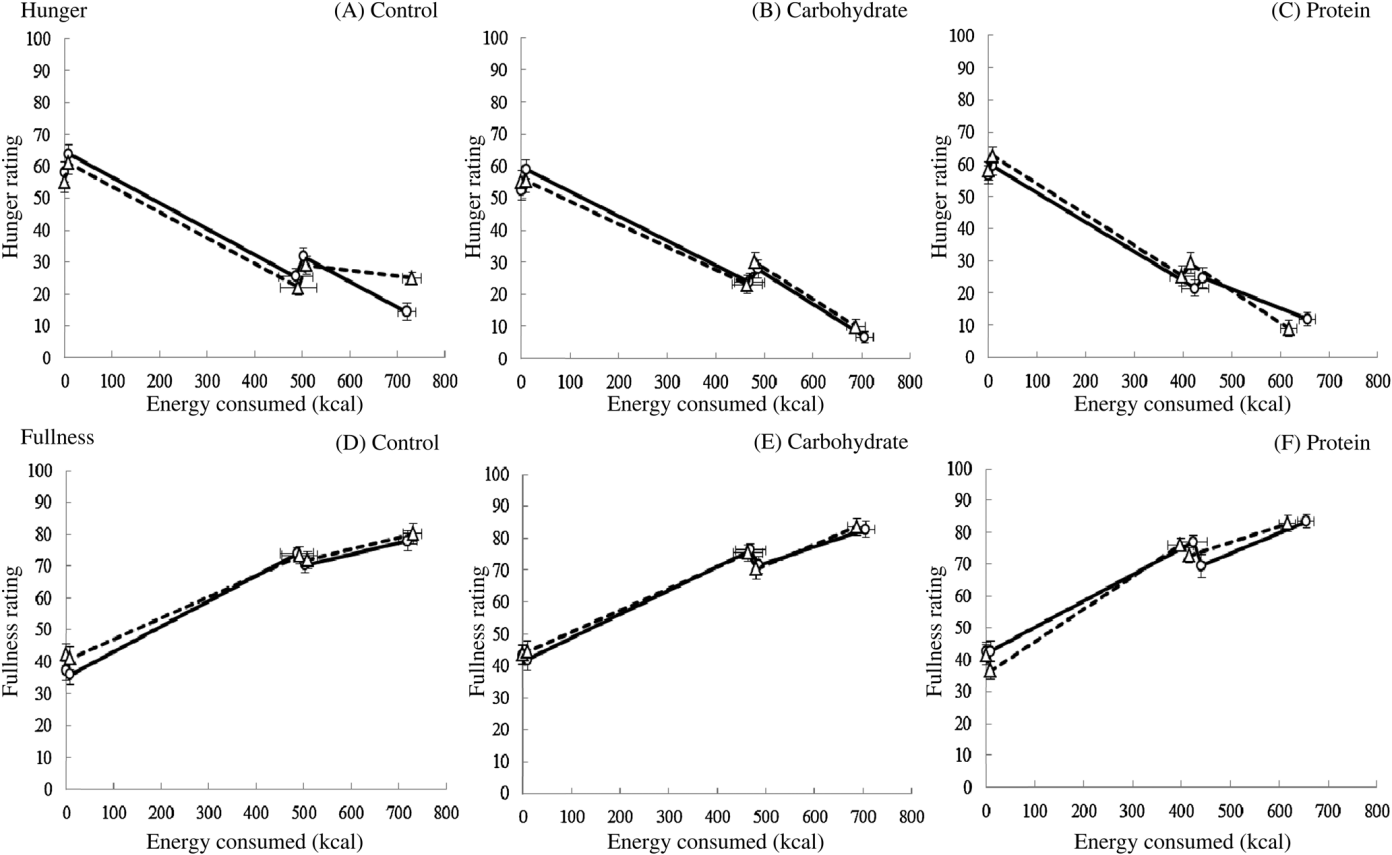
Change in hunger (A, B and C) and fullness (D, E and F) ratings over the duration of an *ad libitum* test meal (energy consumed) following consumption of three versions of soup (low-energy control (A and D); high-energy carbohydrate (B and E); high-energy protein (C and F)) with (- - -) and without (–––) added monosodium glutamate. Values are means, with standard errors represented by vertical bars. See text for statistical analysis. To convert kcal to kJ, multiply by 4·184.

Changes in fullness and hunger during the test meal were examined separately to see whether the nutrient and MSG preload manipulations modified the rate of satiation for the test meal. Over the course of the test meal protein maintained increased satiety (hunger: *F*(2,68) = 15·18, *P* ≤ 0·001; fullness: *F*(2,68) = 4·01, *P* = 0·02) with a significant condition × time interaction (*F*(2,68) = 10·31, *P* ≤ 0·001), suggesting that appetite was most suppressed post-meal after the protein-rich preloads than all other conditions (Fig. 3). Overall, hunger was more suppressed after MSG– soup preloads compared with MSG+ (main effect of MSG: *F*(1,34) = 4·52; *P* = 0·04; *r* 0·34) but this may be driven by the effect of MSG on the carbohydrate-rich condition compared with the protein-rich condition, as a significant condition × MSG × time interaction (*F*(2,68) = 5·39; *P* = 0·007) revealed that the addition of MSG to the carbohydrate-rich soup suppressed hunger less over the course of the *ad libitum* meal when compared with control (*F*(1,34) = 4·15; *P* = 0·05; *r* 0·33) but acted to reduce hunger more in the protein condition when compared with control (*F*(1,34) = 9·77, *P* = 0·004, *r* 0·47; Fig. 3). No significant effects of added MSG were found (*F*(1,34) = 0·43; NS) and no MSG × condition interaction was evident (*F*(2,68) = 1·08; NS).

### Sensory ratings of the preload and test meal

There were no significant differences noted for familiarity (*F*(2,68) = 0·08; NS), pleasantness (*F*(2,68) = 2·93; NS) or sweetness (*F*(2,68) = 2·31; NS) across soup conditions; all soups were deemed familiar, pleasant and marginally sweet (Table 4). MSG+ soups were rated as more salty (*F*(1,34) = 26·26; *P* ≤ 0·001) and stronger tasting (*F*(1,34) = 12·57; *P* = 0·001) than MSG– soups and there was a significant soup × MSG interaction found for savoury assessments (*F*(2,68) = 4·37; *P* = 0·02), with MSG+ control and protein-rich soups deemed more savoury than MSG– but MSG+ carbohydrate soups rated as less savoury than MSG– conditions (Table 4). This may be due to the ambiguous nature of the ‘savoury’ label affecting sensory judgements as trained sensory panels were not tested. The test lunch items were deemed equally familiar across conditions. There was a significant difference in savoury course pleasantness (*F*(2,68) = 5·93; *P* = 0·004), and strength of flavour (*F*(2,68) = 3·16; *P* = 0·05) ratings across conditions, with higher ratings made after protein preload consumption, followed by control and carbohydrate conditions. There were no significant differences in sensory ratings across conditions for the dessert course.

**Table 4.** Visual analogue scale ratings of the sensory characteristics of three versions of soup (low-energy control, high-energy carbohydrate and high-energy protein) with (MSG+) and without (MSG–) added monosodium glutamate (Mean values with their standard errors)

	Low-energy control				High-energy carbohydrate				High-energy protein			
	MSG–		MSG +		MSG–		MSG +		MSG–		MSG +	
Rating	Mean	sem	Mean	sem	Mean	sem	Mean	sem	Mean	sem	Mean	sem
Familiar	70·3	3·6	68·6	3·3	72·3	2·5	69·4	3·5	69·9	3·5	70·1	3·2
Pleasant	60·5	3·4	65·9	1·8	66·9	2·2	65·8	2·7	60·6	3·2	61·1	3·5
Salty	45·4	3·1	50·7	3·2	40·1	2·7	49·9	3·1	41·5	3·6	53·9	3·7
Savoury	61·2	2·4	63·8	2·3	61·3	2·0	57·2	3·2	57·7	3·2	63·7	2·9
Strong	51·5	3·5	56·5	2·6	49·6	2·7	56·4	3·0	49·9	3·6	54·3	3·4
Sweet	55·1	2·6	50·0	3·0	56·7	2·3	57·6	2·6	54·3	2·8	51·8	2·8

## Discussion

The main findings of the study indicate that nutrients received largely as protein in a soup preload allow for an adequate adjustment of energy consumed at a later *ad libitum* meal. Increasing the overall energy content of the test soup mainly with added protein resulted in a greater reduction in lunch intake, and consequently more accurate compensation for this added energy, than was seen when energy was increased by addition of carbohydrate, in line with several recent studies^(^^23^^,^^39,^^40^^)^. Although the predicted enhancement of protein-induced satiety by the addition of MSG was not significant based on intake data, the addition of MSG further improved compensation for added protein energy. However, there were no differences between MSG or specific macronutrient conditions in rated satiety over the course of testing after preload intake.

As predicted, test meal intake after consumption of the protein-enriched preload was lowest followed by the carbohydrate and control conditions consistent with the broader literature, suggesting that protein is more satiating than is carbohydrate^(^^18^^–^^20^^)^. Importantly, compensation became even more accurate when MSG was added to the protein-rich compared with the carbohydrate-rich preload, with this effect strongest in the savoury course of the test meal. This compensation effect was evident despite the relatively small energy difference between low- and high-energy conditions. Indeed, in previous studies compensation effects have only been apparent with larger energy differences between preloads^(^^23^^,^^40,^^41^^)^. This suggests that moderate increases in the energy content of a food through the addition of protein and MSG, for example as a savoury snack, may reduce the likelihood of subsequent overconsumption.

We suggest two possible explanations for how MSG may enhance compensation for energy added as protein. First, the sensory quality generated by the addition of MSG may have made the experience of protein more salient, and so enhanced the satiating effects of protein at the test meal. This idea is supported by recent data showing no significant differences in compensation at a test meal following high-protein and high-carbohydrate preloads when these were matched for thickness and creaminess^(^^28^^)^. Indeed, it may be that the characteristics of MSG are more strongly associated with protein than are the sensory characteristics of thickness and creaminess previously identified and thus act as a more reliable cue for protein-based satiety. Alternatively, this improved compensation of protein in the MSG+ condition could be related to post-ingestive stimulation of gut glutamate sensors^(^^15^^,^^42^^)^ which have been related to enhanced satiety in animals^(^^43^^–^^45^^)^.

An appetising effect^(^^4^^,^^46^^)^ of MSG was seen in both the control and carbohydrate-rich soups with added MSG but was not found in the equivalent protein-rich soup. This may be due to the immediate sensory experience of protein and MSG eliciting lower feelings of hunger in the protein-rich MSG+ condition; however, as *ad libitum* intake of the soup course was not assessed, this remains as speculation. Despite the immediate stimulation of appetite by added MSG in some conditions, no significant differences in hunger were seen immediately after ingesting the soup, in contrast to our recent study^(^^15^^)^. This may be related to volume and hedonic assessments, with participants expecting each soup to be equally satiating due to the equivalent volumes consumed^(^^47^^)^. These predetermined portion sizes may then have influenced hedonic hunger^(^^48^^)^. No difference in appetite was also evident immediately before the *ad libitum* meal 45 min after preload ingestion and may be due to participants responding in anticipation of the meal to be received^(^^49^^)^. Hunger also remained stronger at the *ad libitum* meal in added MSG control and carbohydrate-rich conditions but was suppressed in added MSG protein-rich conditions. Such an effect may be indicative of delayed rebound hunger^(^^40^^)^, as participants consuming added MSG preloads without the accompanying protein may have been anticipating stronger satiety due to the MSG protein cue. However, as a large dose of protein was not delivered, hunger was not as satisfied at the end of the test meal as when no protein cue was present (in no-MSG conditions). But when added MSG was presented in combination with protein, the additional protein cue from MSG acted to reduce feelings of hunger more strongly, suggesting that the added MSG may have increased the salience of this cue as has been found previously^(^^15^^)^ and is evident in infants experiencing umami taste in mother's milk^(^^29^^,^^30^^)^.

The present study also noted that most of the effects of the preload manipulations on lunch intake were evident for the first (savoury) course whereas intake at the dessert course was not affected by preload type. This may be due to the high palatability of this course overriding sensory and energy effects as has been found previously^(^^41^^)^, as sweet appetite relies less on the experience of hunger^(^^50^^)^ and more on the hedonic effects of palatability^(^^51^^,^^52^^)^. Thus consumption of a sweet course may be less suppressed by a previously consumed savoury course^(^^53^^)^, with the critical impact on behaviour being an earlier switch from savoury to sweet courses. There were also a number of limitations in the present design that constrained the conclusions drawn. Due to reformulations of the *ad libitum* main course item, the different versions of the main course may have influenced test meal intake. However, further analyses of intake taking this into account indicated that this was most likely not the case. Similarly, effects of order on *ad libitum* intake indicated that consumption was greater after the first test day but thereafter consumption was in line with the nutrients ingested, indicating that these order effects should not have influenced the sensory–nutrient interactions reported. Initial power analyses indicated that the sample tested would yield adequate power; however, although rated appetite and intake were in the direction predicted they was not found to be significant. This may suggest that a larger sample would be required to assess the more subtle effects of MSG on appetite. It must also be noted that some research has claimed that there can be adverse effects for some consumers when they ingest MSG^(^^54^^)^ although double-blind studies suggest this may be more due to expectation than actual effects of MSG^(^^55^^,^^56^^)^. Further research is warranted to truly understand such findings in human subjects.

Overall, the addition of protein to a soup preload reduced subsequent intake and allowed for more accurate energy compensation at a test meal and this was enhanced by the addition of MSG. However, subjective satiety ratings were not influenced by MSG, the nutrients tested or energy 45 min after preload intake. Further research is required to understand the influence of MSG and protein on sensory and gut responding as well as measures of appetite hormones during and after intake of the preload conditions assessed to gain a more detailed understanding of how sensory–nutrient interactions influence rated appetite and subsequent intake.
